# Neurosteroids Progesterone and Dehydroepiandrosterone: Molecular Mechanisms of Action in Neuroprotection and Neuroinflammation

**DOI:** 10.3390/ph18070945

**Published:** 2025-06-23

**Authors:** Tatiana A. Fedotcheva, Nikolay L. Shimanovsky

**Affiliations:** Laboratory of Molecular Pharmacology, Pirogov Russian National Research Medical University, 1 Ostrovityanova St., Moscow 117997, Russia; shimannn@yandex.ru

**Keywords:** pregnenolone, progesterone, allopregnanolone, dehydroepiandrosterone, inflammation, sigma receptors, PGRMC, PXR, TSPO, BDNF, mPTP

## Abstract

Neurosteroids pregnenolone, progesterone, allopregnanolone, and dehydroepiandrosterone have been actively studied in the last years as candidates for the treatment of neurodegenerative diseases and postinjury rehabilitation. The neuroprotective mechanisms of these neurosteroids have been shown in clinical studies of depression, epilepsy, status epilepticus, traumatic brain injury, fragile X syndrome, and chemical neurotoxicity. However, only the allopregnanolone analogs brexanolone and zuranolone have been recently approved by the FDA for the treatment of depression. The aim of this review was to evaluate whether the endogenous neurosteroids can be used in clinical practice as neuroprotectors. Neurosteroids are multitarget compounds with strong anti-inflammatory, immunomodulatory, and cytoprotective action; they stimulate the synthesis and release of BDNF and increase remyelination and regeneration. In addition to nuclear and membrane steroid hormone receptors, such as PR, mPR, PGRMC1,2, ER, AR, CAR, and PXR, they can bind to GABAA receptors, NMDA receptors, Sigma-1 and -2 receptors (σ1-R/σ2-R). Among these, mPRs, PGRMC1,2, sigma receptors, and mitochondrial proteins attract comprehensive attention because of strong binding with the P4 and DHEA, but subsequent signaling is poorly studied. Other plasma membrane and mitochondrial proteins are involved in the rapid nongenomic neuroprotective action of neurosteroids. P-glycoprotein, BCL-2 proteins, and the components of the mitochondrial permeability transition pore (mPTP) play a significant role in the defense against the injuries of the brain and the peripheral nervous system. The role of these proteins in the molecular mechanisms of action in neuroprotection and neuroinflammation has not yet been clearly established. The aspects of their participation in these pathological processes are discussed. New formulations, such as lipophilic emulsions, nanogels, and microneedle array patches, are attractive strategies to overcome the low bioavailability of these neurosteroids for the amelioration and treatment of various nervous disorders.

## 1. Introduction

With an aging population globally, the burden of diseases of the central nervous system is growing, especially stroke, Alzheimer’s disease, and Parkinson’s disease, thus requiring the creation of new surgical, physiotherapeutic, and drug approaches. The strategies for the prevention, treatment, and rehabilitation of neurodegenerative and neuroinflammatory processes include new surgical approaches [1,2], aerobic physical exercise [3], the use of biofeedback [4], and various pharmacological methods for correcting these pathological conditions.

The modern trend is to use safe natural compounds such as a broad spectrum of polyphenolic compounds [5,6] and other bioactive herbal substances, like DL-3-n-butylphthalide (NBP) [7] or 7,8-dihydroxyflavone [8], which have been shown to suppress neuroinflammation and oxidative stress in humans and animal models.

Among natural compounds, neurosteroids attract extensive attention as candidates for neuroprotectors and anti-inflammatory drugs, especially P4, DHEA, and their derivatives. They are safe, well-tolerated, and, for the most part, have no side effects, except GABAA-mediated side effects like sleepiness, headaches, and dizziness [9]. The side effects related to P4 include water retention, weight changes, and acne due to its possible minimal glucocorticoid, mineralocorticoid, or androgenic activity, respectively, which allows one to use P4 during pregnancy and in reproductive technologies [10]. No side effects were reported for the 1195 traumatic brain injury (TBI) patients of the SyNAPSe trial, and there was only a slight increase in the risk of phlebitis and thrombophlebitis in the patients of the ProTECT III trial. In both trials, very high doses of P4 were continuously administered intravenously for 5 days to TBI patients, with circulating levels of P4 reaching 1 μM, which is higher than the physiological level [11].

The aim of this review was to evaluate whether the neurosteroids pregnenolone (PREG), progesterone (P4), dehydroepiandrosterone (DHEA), and allopregnanolone (ALLO) can be used in clinical practice as neuroprotectors. Many attempts have been made to prove their effectiveness in preclinical and clinical trials, which have shown a moderate improvement in neuropathological parameters such as the cognitive function in depression, stress reduction [12], the reduction in the damaged area during traumatic injuries and reperfusion [13], and pain mitigation [14]. The problem of some studies was the choice of the right dosage, the route of administration, the duration of the study, as well as the outcome measures. PREG has been studied at dosages that vary across a very wide range: from 5 to 800 mg/day. The outcome measures for the evaluation of the effectiveness of the treatment also significantly varied and were not equivalent: for example, some clinical trials used the Aberrant Behavior Checklist with the Irritability subscale; other results were based on self-reported pain. It is really difficult to evaluate the clinical significance of the neuroprotective properties of certain substances since the criteria are often self-reported and based on neuropsychiatric parameters that are hardly measurable or not objective enough.

The molecular mechanisms of the action of neurosteroids are a subject of wide speculation and are still not clearly understood. It was previously shown that GABA and NMDA receptors play a central role in neuroprotection through their allosteric modulation. Recently, the global role of neuroinflammation in different neuropsychiatric disorders has been actively discussed. Alterations in the level of neurosteroids were observed in neuroinflammation-related diseases [15,16,17,18]. The mechanisms of the neuroprotective action of neurosteroids have been extensively studied not only in mammals but also in Zebrafish. Recently, paqr5b, a membrane progestin receptor and a possible receptor for neurosteroids P4, PREG, and ALLO, was identified in a Zebrafish model: Kd for P4 was as low as 5.32 nM; for ALLO, it was 25 nM, and for PREG, it was 28 nM [19]. P4 targets the mPR, which induces the phosphorylation of the GABAA receptor; this leads to the loss of locomotor activity [20]. The same mechanism of activation of the GABA receptor was shown in mammalian cells [21].

The neuroprotective mechanisms of the pharmaceutical substances usually include the antioxidant, anti-inflammatory, neurotrophic, and bioenergetic activities and, more rarely, neurogenesis. In this context, neurosteroids such as PREG, P4, ALLO, and DHEA are promising candidates for clinical use, as all these properties are inherent to them.

In this review, the mechanisms of the neuroprotective effects of the neurosteroids PREG, P4, ALLO, and DHEA are summarized. Special attention is paid to the nongenomic targets of the neuroprotective action of neurosteroids such as P-glycoprotein, BCL-2 proteins, and the components of mPTP.

## 2. Classification of Neurosteroids

Until now, there has been no established classification of neurosteroids (NSs). According to Raciti and Reddy, neurosteroids can be divided into three groups: pregnane NSs (ALLO and allotetrahydrodeoxycorticosterone), androstane NSs (androstanediol and etiocholanone), and sulfated NSs pregnenolone sulfate and dehydroepiandrosterone sulfate (DHEAS) [22,23,24]. According to Tuem, vitamin D is also categorized as a NS [25]. This classification does not assign P4 to any group, which is problematic.

NSs that are synthesized in the brain de novo and exert pharmacologic effects on the neuronal function are defined as neuroactive steroids [26]. All neuroactive steroids are synthesized from their precursor PREG and include PREG itself, its sulfated form, P4, and the latter’s metabolite ALLO, DHEA and the latter’s sulfated form DHEAS [26]. PREG can be converted to P4 or DHEA, and both of them are also neuroactive steroids. In the nervous system, neurosteroids can be formed de novo from cholesterol or from circulating steroid hormones.

Based on these pathways of synthesis, neurosteroids could be classified either as precursors, such as PREG, DHEA, and P4, or metabolites, such as ALLO and sulfated forms of PREG and DHEA [27]. PREG and DHEA can be converted to pregnenolone sulfate and DHEAS by cytosolic sulfotransferases. The sulfation reactions are reversible, and steroid sulfates can be desulfated by steroid sulfatase [28]. The biological effects of sulfated forms and native forms are not yet well established and differentiated. Therefore, it is not necessary to divide sulfated and non-sulfated steroids into two different groups.

Neuroactive steroids could be effective as exogenous treatments [29]. The anesthetic and anticonvulsant actions of P4 have been known since the 1940s, and the antidepressant and anxiolytic effects of ALLO and DHEA have been known since the 1990s [30,31,32,33,34]. The novel opportunities for their clinical application are rehabilitation after stroke injury [35] and the treatment of cocaine addiction [36] and depression [37].

In this review, we discuss the molecular mechanisms of the most promising neurosteroids, namely, P4 and DHEA, and their derivatives in neuroprotection.

## 3. Clinically Approved and Investigational Derivatives of P4 and DHEA in Neuroprotection

The neuroactive steroids PREG, P4, ALLO, and DHEA could be effective as exogenous treatments. Many preclinical and clinical trials have been conducted, which revealed the antidepressant, anesthetic, and antiepileptic effects of P4 and ALLO as well as the neuroprotective effects of ALLO, pregnenolone sulfate, and DHEA in neurodegenerative disorders [26]. Clinically approved and investigational P4 and DHEA derivatives in neuroprotection are listed in the Table 1.

Table 1 demonstrates a wide range of future possibilities for the clinical application of neurosteroids. Three novel drugs have already been approved by the FDA: brexanolone (Zulresso™) and zuranolone (Zurzuvae™) for the treatment of postpartum depression and ganaxolone (Ztalmy™) for the treatment of CDKL5 deficiency disorder. Both are derivatives of ALLO, and their main target is the GABA-A receptor. Zuranolone is under investigation for the treatment of major depressive disorder [52]. Zuranolone has shown more attractive pharmacokinetic parameters than brexanolone due to the possibility of oral uptake and has therefore replaced brexanolone from the market. The procedure for the postpartum depression treatment with brexanolone is very expensive since stationary conditions for intravenous administration are needed [53,55].

The emerging knowledge of the mechanisms of the nongenomically initiated neuroprotective action of P4, DHEA, and their derivatives has been actively discussed in the past decade. Among neurosteroid derivatives, the most attractive one, with regard to neuroprotection, is the ALLO analog zuranolone [52,53], and among DHEA derivatives, these are Triplex (ne3107) and BNN27 [56].

In addition to synthetic neurosteroids, natural endogenous neurosteroids such as P4, PREG, ALLO, and DHEA are still undergoing clinical trials as neuroprotectors in different neurological disorders. Until now, the most promising candidates were DHEA and ALLO.

## 4. DHEA as a Neuroprotector

The horizons for DHEA usage in clinical practice could be much wider than it is today. There is only one FDA-approved drug with DHEA as an active pharmacological substance, which is prasterone for the treatment of dyspareunia [57].

At the same time, there are many dietary supplements with a daily dose of 50 mg for the improvement of erectile dysfunction, infertility, muscle strength, aging skin regeneration, moderate depression, cardiovascular system protection, and many other conditions. At this dosage, dietary supplements with DHEA have positive cognitive antiaging effects in menopausal women [58] and elderly men [59].

The anti-stress effects of DHEA are commonly perceived as anticortisol effects [60]. DHEA exerts its biological action through the activation of various receptors, including both nuclear receptors, such as androgen receptor (AR), estrogen receptor (ER), progesterone receptor (PR), constitutive androstane receptor (CAR), peroxisome proliferator activated receptor (PPAR), pregnane X receptor (PXR), SIGMA-1R (σ1-R), and ion channel receptors, such as GABA and NMDA receptors. DHEA also directly binds DHEA-specific G-protein-coupled receptors in endothelial cells [61]. Although the mechanism of action of DHEA is not fully understood, it is known that the hormone can exert its effect on target cells indirectly, after its conversion to androgens and/or estrogens, and directly, by acting mainly as a neurosteroid [62,63].

DHEA and DHEAS predominantly act as noncompetitive antagonists of the GABAA receptor. DHEAS has more potent antagonistic effects than DHEA [18]. DHEAS is a positive allosteric modulator of NMDA receptors. The mechanisms of the neuroprotective action of DHEA include anti-inflammatory, immunomodulatory, and regenerating activities as well as the maintenance of the redox homeostasis of neuronal cells. The receptor-mediated effects of DHEA are the inhibition of NMDA-induced neurotoxicity and the antagonism of corticosterone stress-induced neurotoxicity. The anti-inflammatory action of DHEA is due to the differentiation of Th2, the inhibition of NF-kB activation, and direct anti-IL-6 receptor activity [64,65,66].

DHEA and other neurosteroids can directly regulate the synthesis of neurotrophic proteins (Table 2). Neuroregeneration, particularly proliferation and maturation, requires different neurotrophic agents, such as the nerve growth factor (NGF), neurotrophin-3 (NT-3), neurotrophin-4 (NT-4), BDNF, and others.

The neuroprotective properties of DHEA depend on its concentration near the target. DHEA can exert rapid anxiolytic and anticonvulsant effects, but at high dosage, it can exhibit sedative and hypnotic properties [67].

Another target activated by DHEA is the non-opioid intracellular receptor Sigma-1 (Sig-1R (σ1-R)), by which DHEA normalizes calcium homeostasis. Another target of DHEA, CAR (constitutive androstane receptor), also belongs to the nuclear receptor superfamily (subfamily 1, group I, member 3, also known as NR1I3). CAR counteracts the key signaling pathways involved in energy metabolism, xenobiotic metabolism, and bilirubin metabolism [68]. The activation of CAR in human hepatocytes by DHEA results in the induction of cytochrome CYP2B6 [69]. However, CAR signaling is poorly understood, and new knowledge regarding the involvement of this receptor in neuroprotection may soon emerge. Future prospects lie in the development of new DHEA dosage forms with improved bioavailability because DHEA itself has a low oral bioavailability of about 3–10% [56]. When administered by the percutaneous route, DHEA has higher bioactivity than when it is given orally [70].

An important mechanism of the neuroprotective action of DHEA is the stimulation of BDNF synthesis since DHEA directly binds to the BDNF receptor TrkB (tyrosine kinase B). Like other neurosteroids, DHEA can significantly increase the synthesis of nerve growth factors (Table 2).

**Table 2:** Effect of neurosteroids PREG, DHEA, P4, and ALLO on the expression of nerve growth factors.

Nerve Growth Factor	PREG	DHEA	P4	ALLO
BDNF	normalizes the BDNF level in dopamine-depleted striatum [71]	increases the expression of BDNF in rats [72]	markedly mitigates the increased level of mature BDNF [73]	mitigates the decreases in truncated BDNF [74]
GDNF	no specific data on the direct effect on GDNF are available	no specific data on the direct effect on GDNF are available	the intracellular content of GDNF is not affected by progesterone treatment [75]	promotes neurogenesis and neuroprotection and modulates neurotrophic pathways indirectly through its influence on neuronal and glial cell activity [24]
CNTF	no specific data on the direct effect on GDNF are available	DHEA’s ability to enhance neural stem cell proliferation and neurogenesis may create cellular environments where CNTF is upregulated, as CNTF supports neuronal survival and differentiation [76,77]	P4 inhibits CNTF expression in cultured C6 astroglioma cells. Progesterone treatment also reduces CNTF expression in the amygdala and decreases immobility time in female CNTF+/+ but not in CNTF−/− mice [78]	no specific data on the direct effect on CNTF are available

## 5. Progesterone as a Neuroprotector

Unlike DHEA, P4 is used in different dosage forms for a wide range of procedures: from in vitro fertilization to the treatment of hormone-dependent tumors [79]. However, it has never been used in the treatment of nervous diseases, although there are all the prerequisites for this.

Reddy discovered long ago that P4 has an anxiolytic effect [80], and other studies demonstrated its sedative and anticonvulsant effects [81,82]. P4 can produce sedative-like effects in both men and women [83].

The gender differences in the P4 treatment of catamenial epilepsy have been extensively studied by Herzog and coauthors [84]. They showed that P4 plays a central role in the development of catamenial epilepsy. Unlike estrogen, the role of P4 becomes clearer when consistent anticonvulsant and antiepileptic properties in animals and humans are taken into account. Multicenter clinical trials have been conducted in women with epilepsy [85,86]. It has been assumed that perimenstrual catamenial epilepsy is associated with the withdrawal of anticonvulsant neurosteroids. However, the conclusions and end points of the clinical trials with P4 have demonstrated moderate effectiveness in catamenial epilepsy and some endocrine side effects [80]. The side effects could be due to the binding of P4 with other sex steroid nuclear receptors. Thus, more specific membrane P4 receptor selective modulators are needed to exert only P4-dependent neuroprotective action.

P4 realizes its neuroprotective action predominantly through nongenomic effects and membrane receptors since its neuroprotective effectiveness has been demonstrated within seconds in different models of traumatic brain injury.

The main signaling pathways of P4 involve extracellular signal-regulated kinase (ERK), cAMP/protein kinase A (PKA), protein kinase G (PKG), Ca^2+^ influx/protein kinase C (PKC), and phosphatidylinositol 3-kinase (PI3 K)/Akt [87].

The neuroprotective action of P4 is brought about through membrane-associated P4 receptors (PGRMC1/2), membrane progesterone receptors (mPRs), NMDA and GABAA receptors, Sigma-1/2 receptors, and the classical nuclear P4 receptors [88]. Recently, α/β hydrolase domain-containing protein 2 (ABHD2) has been proposed as a new receptor of P4 involved in several other nongenomic P4 actions [89]. Later, this role of ABHD2 was disproved, as no evidence has been found for the direct binding of P4 to the purified recombinant ABHD2 protein [90].

Castelnovo showed that the neuroprotective action of P4 in a Parkinson’s disease human cell model is brought about the membrane progesterone receptor α (mPRα/PAQR7) [91], which is in good agreement with the previously obtained results since mPRs have been reported to mediate the neuroprotective effects of progestogens in different neuronal cell line models [92].

Membrane receptors are involved in the anti-inflammatory and immunosuppressive activities of P4 [93,94]. P4 also inhibits nitric oxide production and the expression of toll-like receptors by macrophages and promotes Th2 differentiation in vitro [93].

Knowledge of the molecular mechanisms of the anti-inflammatory and immunomodulatory effects of P4 may open up new pathways in neuroprotection and the restoration of neuroplasticity in the central nervous system.

Progestins can restore the chemosensitivity to drugs by inhibiting P-glycoprotein and PXR. However, the mode of action on PXR is poorly understood and controversial, as at the same time, PXR stimulation leads to the release of BDNF, which is responsible for neuroprotection. Different regulators, such as microRNA, can modulate hormonal effects on the release of nerve growth factors [95,96]. It was shown that the stimulation of GDNF release from glial cells appears as a potential mechanism through which P4 exerts its neuroprotective effects. Treatment with P4 (10 µM) for 48 h resulted in a significant elevation of GDNF secretion from C6 glioma cells, which remained elevated up to 72 h. However, the intracellular content of GDNF and cell numbers were not affected by P4 treatment [75], as shown in Table 2.

The Singh laboratory has previously demonstrated that PGRMC1 selective knockdown completely abolishes the ability of P4 to increase BDNF release. It has been suggested that the classical PR and PGRMC1 are critical, although perhaps not exclusive, mediators of the effects of P4 on cell viability [96].

During injury, the concentration of P4 in the brain increases. P4 is responsible for the formation of new myelin sheaths after nerve injury. Nuclear PR was responsible for myelin synthesis since this effect could be mimicked by the very selective PR agonist promegestone, and it was no longer observed in cerebellar slices prepared from PR knockout mice. Thus, progestins as neuroprotective and remyelinating agents are a promising strategy in the treatment of neurodegenerative disorders [92].

In this context, progestins can be useful drugs in the treatment of demyelinating diseases such as multiple sclerosis (MS), but they should not be used in the treatment of meningioma [97] because P4 increases the number of oligodendrocytes expressing the myelin basic protein and the 2′,3′-cyclic nucleotide-3′-phosphodiesterase [98]. However, clinical trials with P4 in the treatment of MS, in the pathogenesis of which demyelination plays a major role, have not been successful. Only estriol has demonstrated more or less pronounced effects, positively changing the course of MS [99].

Thus, P4 has mostly anti-inflammatory and immunosuppressive action, which also improves the profile of the synthesis of nerve growth factors.

## 6. Pregnenolone as a Neuroprotector

PREG is synthesized from cholesterol and is a precursor of all other steroids. PREG and its stereoisomer ALLO are among the most potent neurosteroids in positively and allosterically modulating GABAA receptors [100].

ALLO and PREG, neurosteroids synthesized from P4 in the brain, the adrenal gland, the ovary, and the testis [101], have been implicated in a range of neuropsychiatric conditions, including seizure disorders, post-traumatic stress disorder, major depression, postpartum depression, premenstrual dysphoric disorder, chronic pain, Parkinson’s disease, and Alzheimer disease [26,102].

PREG has been intensively studied as a drug for the treatment of autism, marijuana dependence, and alcohol use disorder; in addition, positive results have been found for the treatment of major depressive disorders [103]. Eltanolone, a synonym for PREG, is under investigation in clinical trial NCT02603926 (Treatment of Fragile X-Associated Tremor/Ataxia Syndrome (FXTAS) with Allopregnanolone). The results from other clinical trials are also reliable: at a dose of 500 mg/day, PREG improved cognitive function in schizophrenia [104]; at a dose of 50 mg/day, it reduced the severity of negative symptoms in recent-onset schizophrenia and schizoaffective disorder, especially among patients who were not treated with concomitant mood stabilizers [105]; and at doses of 500 mg/day for 12 weeks, it had a positive effect on the rate of dipolar depression remission [106]. In the treatment of autism, PREG was modestly effective and well-tolerated [107]. A pilot, open-label, 12-week trial was undertaken, which included twelve subjects of a mean age of 22.5 ± 5.8 years. PREG yielded a statistically significant improvement in the primary measure, namely, Aberrant Behavior Checklist (ABC)—Irritability [from 17.4 ± 7.4 at the baseline to 11.2 ± 7.0 at 12 weeks (*p* = 0.028)].

In some clinical trials where doses of 300 and 500 mg per day orally were used, PREG showed no effectiveness. It was believed that the low effectiveness of the compound is due to its poor bioavailability. However, recently, in a pilot study on cocaine users, good absorption was demonstrated when PREG was taken orally: specifically, 2 h after taking the drug, there was a significant and stable increase in the level of PREG in the blood plasma [108]. At higher doses, i.e., 500 and 800 mg capsule by mouth, daily for 7 days, PREG showed positive results in the treatment of back pain [46]. This also suggests that previous clinical trials used exceedingly low doses of PREG to demonstrate the significant efficacy of this neurosteroid.

Whether PREG has its own receptors or implements remains to be studied. Another important task is to determine the level and activity of enzymes involved in the metabolism of PREG.

PREG, like P4, is an anti-inflammatory neurosteroid. It inhibits pro-inflammatory neuroimmune pathways in the periphery and in the brain, independent of GABAergic mechanisms [109]. Both P4 and PREG show promise in the treatment of migraine. Migraine attacks are often accompanied by low levels of PREG [110]. During pregnancy, migraines become less severe because P4 levels increase [111].

## 7. Allopregnanolone as a Neuroprotector

Among all neurosteroids, ALLO is historically considered not as a steroid hormone but exclusively as a neuroactive steroid and a neuromodulator [112]. It is the only neurosteroid that has already been used as an antidepressant, known as ZULRESSO^®^ (brexanolone). According to the target-based classification of drugs, brexanolone is a GABA receptor modulator [113].

ALLO showed neuroprotective effects, together with an influence on neuronal signaling, decreasing the axonal damage and restoring the myelin gene expression by oligodendrocytes, which protected against injury [94]. In addition to myelin-stimulating activity, ALLO upregulated BDNF gene expression by the potentiation of GABAergic neurotransmission and classical PR, while mPRs and PGRMC1 were involved in the release of BDNF [96].

Like other neurosteroids, ALLO belongs to the allosteric positive regulators of the GABA-R protein group, and this can be an explanation for its calming neuroprotective effects [114].

In March 2022, the 3β-methyl synthetic analog of ALLO, ganaxolone (ZTALMY^®^; Marinus Pharmaceuticals), was approved to treat seizures caused by a rare genetic disorder associated with cyclin-dependent kinase-like 5 deficiency in patients 2 years of age and older [115]. It has oral bioavailability and acts as an anxiolytic, a sedative, and an anticonvulsant drug by enhancing the inhibitory effects of GABAA receptors.

Recently, phase 2 of the REGEN-BRAIN clinical trial (NCT04838301, https://clinicaltrials.gov/study/NCT04838301 (accessed on 11 May 2025)) was started, with ALLO as a neuroprotective and regenerative drug for the treatment of Alzheimer’s disease. The study aims to validate the previous findings that indicate that ALLO may exert both regenerative and neuroprotective effects on the structure of and connectivity in the brain of Alzheimer’s patients [116].

## 8. New Molecular Mechanisms of the Neuroprotective Action of Neurosteroids

### 8.1. The Mechanism of Anti-Inflammatory Action

While the action of NSs has been previously explained by the allosteric regulation of NMDA and GABA receptors, now, it is clearer that there are more specific targets of their neuroprotective action. Evidence has appeared of their binding and regulation of signaling through sigma receptors, PXR, and mPTP. Since inflammation has recently been found to have the main role in the pathogenesis of almost all psychiatric diseases, the neuroprotective properties of neurosteroids should be considered as anti-inflammatory. Neurosteroids regulate both pro- and anti-inflammatory signaling cascades. Deficits in neurosteroids cause an activation of pro-inflammatory pathways, which disrupt brain networks [109].

Neuroinflammation develops due to an imbalance of the neurotransmitters norepinephrine, dopamine, acetylcholine, and glutamate, the activation of the hypothalamic–pituitary–adrenal axis and the release of cortisol, and prolonged oxidative and nitrosative stress, leading to neuronal apoptosis [117]. Neurosteroids regulate all these processes [103]. In this context, neurosteroids can also be considered as anticortisol substances.

DHEA, P4, ALLO, and PREG effectively downregulate IL-6, TNFα, and IL-1b [56,66,118], inhibit the Nrf2/ARE signal pathway [119], and upregulate anti-inflammatory mediators, such as IL-10, BDNF, and CX3CL1 [109].

One of the urgent tasks of current investigations is to clarify the precise mechanisms of the anti-inflammatory action of neurosteroids. It has been suggested that neurosteroids reduce neuroinflammation not only by the regulation of IL-1β, IL-6, TNFα, interferon-γ, and CX3CL1/2/10 but also by inhibiting the release of glutamate and nitric oxide by microglia [120].

Neurosteroids have no pro-inflammatory activities and mostly repress excessive inflammatory signaling [109]. Sex steroid hormones, unlike neurosteroids, can cause pro-inflammatory stimuli to evoke a rapid immune response to injury. In this context, androgens and estrogens can stimulate inflammatory responses [121,122]. In activated microglia, the synthesis of ALLO from PREG increases to a greater extent than the synthesis of androgens and estrogens from PREG. There is a significant difference between the actions of androgens and estrogens since female predominant microglia function as M1 phenotype microglia, with inflammation, neurotoxicity, and immune activation. Male predominant microglia function as M2 phenotype microglia, with wound-healing, neuroprotection, and immunosuppression [121].

De novo microglial neurosteroidogenesis appears as a compensatory mechanism after injury and subsequent acute inflammation. Human microglia produce PREG and ALLO in response to oxidative stress to support microglia’s survival [120]. Selective TSPO ligands, known to increase neurosteroid production [123], promote the establishment of an anti-inflammatory phenotype of activated microglia [124]. This is strong evidence that neurosteroids have cytoprotective, immunomodulating, and anti-inflammatory modes of action.

### 8.2. Sigma-1 Receptor (σ1-R) and Sigma-2 Receptor (σ2-R) Signaling

The molecular mechanisms of the neuroprotective action of pregnane neurosteroids can differ from those of androstane neurosteroids in the context of their different binding affinities to σ1-R and σ2-R. σ1-R and σ2-R are located in the endoplasmic reticulum and act as chaperones. σ1-Rs modulate neurotransmitter and calcium signaling by interacting with inositol triphosphate [125]. σ2-Rs form a trimeric complex with PGRMC1 and the low-density lipoprotein receptor (LDLR), and this intact complex is required for the efficient uptake of lipoproteins such as LDL and apolipoprotein E (apoE) [126]. Targeting this complex may represent an effective strategy for reducing the level of lipoproteins relevant to the development of atherosclerosis.

The modulation of PGRMC1/2 and sigma receptors may be one of the main mechanisms of the neuroprotective action of NSs since these receptors regulate the signaling of steroid hormones [127]. There are completed and ongoing clinical trials of the treatment of Alzheimer’s disease with the modulator of the component 1 (PGRMC1)/σ2-R complex, σ2-R receptor antagonist, and oral small molecule CT1812, which is capable of penetrating through the blood–brain barrier [128]. CT1812 interferes with the binding of Aβ oligomers to neurons by displacing them into the cerebrospinal fluid. The outcomes of these trials were significant exposure-dependent increases in Aβ oligomers and tau phosphorylation as well as a decrease in the level of GSK3β, which is consistent with the potential upstream modulation of tau kinases. The endpoints and outcomes were measured in six clinical trials of CT1812 by neuroimaging and evaluation of cognitive function, EEG biomarkers, cerebrospinal fluid biomarkers, and synaptic effects [129].

Until now, there was no experimental evidence of the functional significance of PGRMC1 as a monomer protein since PGRMC1 has been studied mainly in cancer animal models. PGRMC1 contributes to the progression of proliferation and resistance to chemotherapy of different solid tumors [130,131].

Almost nothing is known about the role of PGRMC2 in neuroprotection. This aspect demands further research. Recently, it was shown that PGRMC2 was elevated in different brain cells after ischemic stroke in male mice. At the same time, the intraperitoneal injection of PGRMC2 activator CPAG-1 reduced the size of the infarct, inhibited astrocyte and microglial activation, and improved sensorimotor deficits after ischemic stroke [132].

All psychoactive substances modulate sigma receptors as agonists or antagonists. For neuroprotection processes, σ1-R agonists and σ2-R antagonists are preferred.

#### 8.2.1. Sigma-1 Receptor (σ1-R)

Previously, it was considered that the Sigma-1 receptor (Sig-1R or σ1-R) is an endoplasmic reticulum (ER) protein located in the mitochondria-associated ER membrane, the interface between the ER and mitochondria. However, upon stimulation by agonists or stressors, σ1-R can translocate to the plasma membrane to interact with ion channels, receptors, and kinases. Thus, σ1-R has no strict localization in the ER. In addition to its ability to translocate to the plasma membrane to interact with ion channels and other receptors, σ1-R is also found in the nucleus, where it can work as a transcription factor. σ1-R can interact with different signaling molecules in the cytosol as well [133]. However, the dominant location is a specific domain of the mitochondria-associated ER membrane. σ1-R forms ion channels with voltage-dependent anion channels (VDACs), regulating the ER stress and calcium homeostasis. It regulates Ca^2+^ transport from the ER into the mitochondria and indirectly regulates mitochondrial ATP synthesis. These interactions indicate that σ1-R ligands can modulate mPTP opening. In this context, NSs can act as σ1-R ligands and, therefore, as mPTP opening regulators.

NSs bind to σ1-R, as demonstrated in in vitro and in vivo binding assays. P4 is generally considered a putative σ1-R antagonist, while other NSs are often considered to be putative σ1-R agonists [134]. Recently, the crystallographic structures of σ1-R bound to two NSs, the putative antagonist P4 and the putative agonist DHEAS, were obtained.

Most studies have demonstrated the neuroprotective role of σ1-R agonists. At the same time, whether σ1R ligands exert agonistic or antagonistic action remains controversial. On the one hand, σ1-R activation ameliorates neurodegenerative disease by balancing ion homeostasis, regulating ER stress and oxidative stress, and promoting the expression of the neurotrophic factor and nerve remodeling. On the other hand, another study revealed that the inhibition of σ1-R plays a significant role in improving neuralgia by suppressing neuronal hyperactivity. The functions of Sig-1R under some pathological and normal conditions can differ. σ1-R inhibitors suppress pain sensitivity and ameliorate opioid tolerance caused by long-term application by regulating the activity of opioid receptors. In contrast, stimulating σ1-R induces hyperalgesia by enhancing glutamate release and inhibiting the GABAA receptor.

The preferred action of NSs in a particular pathology, σ1-R-agonistic or σ1-R-antagonistic, can depend on the concentration of neurosteroids and the time of exposure. The classical pharmacological mode of action can be realized in this modulation when the low doses of an agonist are stimulatory and higher doses are inhibitory. It has been proposed that, for neuroprotection and cardioprotection, σ1-R activators are needed. σ1-R agonists, including the endogenous neurosteroid DHEA and the selective serotonin reuptake inhibitor fluvoxamine, have shown strong cardiac protection and antidepressant effects [135].

In addition, σ1-Rs are involved in the release of neurotransmitters, inflammation, synaptogenesis, and neuroplasticity. Neuroplasticity is an important process in neuropsychiatric diseases. PREG and DHEA may produce beneficial effects in some neuropsychiatric disorders, such as σ1-R agonists. The studies demonstrated that the sulfated form of DHEA attenuates phencyclidine-induced cognitive loss in mice [136], whereas P4, like testosterone, inhibits σ1-R signaling [137], which demonstrates the difference between the action of pregnane and that of androstane neurosteroids. Agonists for the σ1-R—DHEA and ALLO elicit nociceptive responses, which can be reversed by the antagonist P4. σ1-Rs are involved in supporting the homeostasis balance between the antagonist P4 and the agonist ALLO [137].

DHEA, DHEAS, and PREG are all σ1-Rs agonists, while P4 is an antagonist. PREG (30 mg) and DHEA (200 mg) have been shown to improve cognitive impairment and clinical symptoms, especially in schizophrenia and psychotic depression [138]. DHEAS significantly attenuated phencyclidine-induced cognitive impairment in mice, and this effect was antagonized by co-administration of the σ1-R antagonist NE-100 [138].

Some nonsteroidal σ1-R agonists, such as fluvoxamine, donepezil, and pridopidine, may be repurposed for the treatment of several neurodegenerative diseases; thus, pridopidine has already shown promising results in preclinical studies and clinical trials [139].

The molecular mechanisms by which NSs modulate σ1-R activity are still unclear. DHEA, as a Sig-1R agonist, has been extensively studied. The stimulation of σ1-R with DHEA was found to improve cognitive functions through the activation of calcium/calmodulin-dependent protein kinase II (CaMKII), PKC, and kinase and the induction of the Akt/GSK-3β/β-catenin pathway [140].

Owing to its neuroprotective effects, σ1-R has now become a breakthrough target for alleviating Alzheimer’s disease and other neurodegenerative diseases [141].

#### 8.2.2. Sigma-2 Receptor (σ2-R) or TMEM97

**Figure 1:**
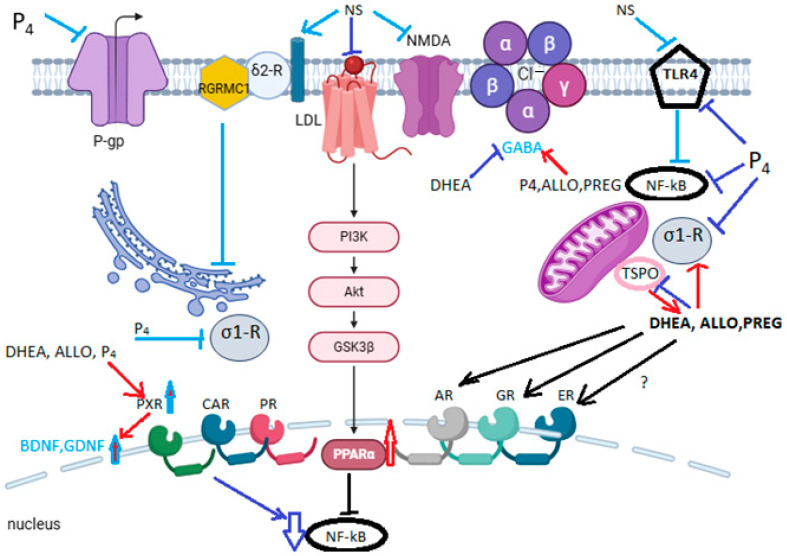
Neurosteroid targets in neuroprotection. Note: NSs bind to the plasma membrane receptors NMDA, GABA, σ2-R/PGRMC1/LDLR complex, and TLR4. P4, ALLO, and PREG are positive regulators of GABA, and DHEA is a negative regulator of GABA. NSs activate PPARa through the σ2-R/PGRMC1/LDLR complex, which leads to NF-kB inhibition in the nucleus. DHEA, ALLO, and P4 activate PXR in the nucleus, which also leads to NF-kB inhibition and, at the same time, increases the synthesis of BDNF and GDNF. P4 inhibits σ1-R signaling, while DHEA, ALLO, and PREG activate σ1-R signaling. TSPO increases NS synthesis and is overexpressed during injury. The participation of the nuclear steroid receptors CAR, AR, GR, and ER in the neuroprotective action of NS is not clear. The figure was created using Biorender (https://biorender.com/).

The ligands of σ2-R (commonly its antagonists) are claimed to be neuroprotective in Alzheimer’s disease, while its agonists are reported to be cytotoxic in cancer. σ2-R is an important biomarker of tumor cell proliferation. The reason for these opposing properties remains unknown [142]. σ2-R is a protein called TMEM97, and it forms a trimeric complex with PGRMC1 and the LDL receptor, which increases the rate of internalization of lipoproteins (Figure 1).

The modulators and active antagonists of σ2-R are being investigated as candidates in the treatment of Alzheimer’s disease since they affect lipoprotein homeostasis. Thus, the selective σ2-R antagonist DKR-1677 was found to be highly neuroprotective after traumatic injury [143]. More recently, this compound was shown to be neuroprotective in ischemia-induced retinal ganglion cell degeneration [144,145].

Another small molecule, CT1812 (Elayta™), an antagonist of σ2-R, is being tested for the treatment of Alzheimer’s disease in Phase 2 clinical trials [146,147]. CT1812 selectively displaces Aβ oligomers, thereby preventing them from binding to neuronal synapses and mitigating downstream toxicity [148]. σ2-R antagonists have also shown promising results in different mouse models of Alzheimer’s disease, traumatic brain injury, and alcohol abuse by counteracting the impaired synaptic function associated with the Aβ1-42 oligomer [149].

Thus, different effects of neurosteroids are observed on sigma receptors. However, since they are chaperones, their regulation should strongly depend on the concentration of neurosteroids. Despite the different effects of DHEA and P4 on sigma receptors, both are neuroprotectors. The role of sigma receptor subtypes remains to be studied, but there is strong evidence that σ2-R regulates cell functions via its coreceptor PGRMC1. σ2-R has been shown to be closely associated with key proteins to exert its functions, including PGRMC1 and LDLR, as shown in Figure 1. Ongoing studies have attempted to elucidate the functions of each protein separately, but σ2-R and PGRMC1 work together and are colocalized and coexpressed; their regulation of amyloid-β binding is obvious [150].

The consideration of the σ2-R/PGRMC1 complex as a key regulator of Aβ1-42 oligomer and apoE accumulation in the cell has led to the conclusion that it may be of clinical significance in the treatment of Alzheimer’s disease. Patients with traumatic brain injury are at an increased risk of developing chronic neurodegenerative diseases, including Alzheimer’s disease. Altered production and clearance of apoE and beta-amyloid (1–42) are involved in the pathogenesis of neurodegeneration and dementia [151]. Disrupting the binding of beta-amyloid (1–42) with synaptic receptors is a promising therapeutic strategy as the sigma-2/PGRMC1 protein is a critical receptor that mediates more than 90% of Aβ oligomer binding to neurons and their downstream synaptotoxic effects [152].

NSs significantly modulate σ2-Rs; among these, P4 binds PGRMC1 directly with high affinity (Kd of about 35 nm) [153]. PGRMC1 functioning is tissue-dependent. It can promote cytoprotection in the brain and the heart after injury [79]. As it was shown on cultured neurons, the neurotrophic activity of PGRMC1 is realized with the involvement of MAP kinase and Akt signaling [154].

PGRMC1 was also shown to interact with MDR-linked targets of progestins [93]. The role of PGRMC1 in drug efflux and P-glycoprotein activity and expression, as well as the role of PXR, should be evaluated in animal models.

Until now, there were no established constants of binding of other NSs to PGRMC1 or σ2-R, and there were no data about the inhibitory or stimulatory effects of NSs on the signaling of this complex. In this context, further research is needed to evaluate the influence of NSs on lipoprotein uptake mediated by the σ2-R/PGRMC1 receptors.

### 8.3. PXR-Mediated Cytoprotection and BDNF Synthesis

PXR is a transcription factor (a nuclear receptor) activated by a wide range of chemicals that have no common structural features. PXR is considered to be a transcriptional regulator of CYP3A induction by xenobiotics [155]. PXR regulates the efflux and elimination of xenobiotics and, therefore, is predominantly expressed in the liver and the intestine. PXR is responsible for the transport of xenobiotics and is activated during drug resistance [156].

NSs promote the expression of nuclear receptors, including PXR and liver xenobiotic receptors (LXRs) [157]. PXR/LXR can modulate the synthesis of steroids and the degree of cellular stress, thereby contributing to anti-inflammatory and neuroprotective actions. The interaction of NSs with PXR/LXR can also promote the synthesis and signaling of BDNF [29]. The activation of PXR is considered one of the mechanisms of neuroprotection [158].

DHEA also acts as an activator of PXR. The activation of PXR by high doses of DHEA in rodents induces the expression of the gene CYP3A, which leads to increased phase 1 metabolism, and this mechanism can protect the neurons from toxic compounds. The activation of PXR leads to the synthesis of BDNF; so, DHEA and other NSs have a neuroprotective effect in mammals [72].

PREG, P4, ALLO, and DHEA were shown to induce BDNF synthesis and release, but little is known about their influence on other growth factors such as NGF, GDNF, NT-3, and NT-4. Table 2 summarizes the data on the action of neurosteroids on the synthesis of key nerve growth factors.

### 8.4. TSPO-Mediated Neuroprotection

The abovementioned receptors σ1/2-R, PGRMC1, and PXR are the novel targets for neuroprotection and are the regulators of neurosteroid biosynthesis. Another very important and possibly key regulator of the pharmacological action of neurosteroids is the translocator protein (TSPO), an 18-kDa protein located in the outer membrane of mitochondria, previously known as the peripheral benzodiazepine receptor. It was experimentally proven that PXR may be located upstream of TSPO, acting as a homeostatic regulator involved in neurosteroidogenesis [157]. The main function of TSPO is the transport of cholesterol. TSPO can be considered a component of mPTP. Recently, a functional interaction between TSPO and σ2-R and their colocalization was confirmed [159]. The role of TSPO is controversial. Since it is overexpressed during neuroinflammation, it was believed that the suppression of its expression has an anti-inflammatory and protective effect. TSPO is a commonly used marker of neuroinflammation in different studies since its expression has been previously found to be increased in several human diseases in post-mortem brain tissues and in animal models of neuroinflammation and neurodegeneration [15].

In the past decades, several classes of TSPO ligands that have anti-inflammatory and neuroprotective effects have been identified in both in vitro and in vivo models.

TSPO can be elevated in mental disorders, such as major depression. Moreover, preclinical and early clinical studies showed that TSPO ligands can have antidepressant and anxiolytic properties, promoting the endogenous synthesis of neurosteroids. Compounds that enhance GABAergic neurotransmission, such as neurosteroids and TSPO ligands, which may also exert anti-inflammatory properties in conjunction with immunomodulators such as C1q, may open up new avenues for the treatment of psychiatric disorders [160,161].

TSPO ligands are being investigated as therapeutic agents for Alzheimer’s disease, Parkinson’s disease, multiple sclerosis, neuropathic pain, and anxiety disorders. TSPO increases the synthesis of neuroprotective steroids, decreases neuroinflammation, limits the opening of mPTP, and reduces the generation of reactive oxygen species [162].

In addition to exogenous steroid administration, the enhancement of endogenous production of NSs by targeting TSPO has been proposed to restore the level of pathologically altered NSs [15].

The mechanisms of the protective effect of TSPO inhibitors are being actively studied [163]. A number of TSPO-specific agonists, antagonists, and other ligands have already been synthesized. One TSPO ligand, etifoxine, is also currently used in clinical practice to treat anxiety, with a minimal side effect profile [164].

The anti-inflammatory and cytoprotective effects of TSPO ligands belonging to the class of N,N-dialkyl-2-arylindol-3-ylglyoxylamides (PIGA, N,N-dialkyl-2-arylindol-3-ylglyoxylamides) are known. The protective effect of PIGA was shown to be neutralized by the cotreatment of cells with the PREG synthesis inhibitor SU-10603, which indicates the participation of neurosteroids, in particular PREG, in the protective mechanism of these TSPO ligands [165].

TSPO has recently been identified as a potential biomarker of neurodegeneration since its expression increases with inflammation and neurodegeneration associated with Alzheimer’s disease, HIV encephalitis, and MS. Given the known anti-inflammatory and antioxidant properties of DHEA, TSPO may be a key protein in the maintenance of DHEA levels [166].

Recent data indicate that TSPO expression is adaptive, increases in acute inflammation, improves mitochondrial biogenesis, reduces oxidative stress, and triggers neurosteroid synthesis. In chronic inflammation, the persistent activation of the protein leads to degenerative processes, apoptosis, and ROS production and is clinically manifested in the same way as in autoimmune neurodegenerative processes. TSPO is a key regulator of neurosteroid synthesis [160]. Neurosteroids can also be TSPO ligands.

TSPO, like inflammatory cytokines, plays a key role in inflammation, being cytoprotective in acute reactions and having negative effects in chronic pathological processes. That is why it is extremely important to take into account the nosology (ischemic stroke or neurodegenerative diseases) and the time of exposure in future clinical studies of neurosteroids. In the first condition, the time of exposure should not be long, and the dosage should be high. TSPO, as a direct participant in steroid biosynthesis, can be damaged due to hereditary pathologies; in this case, the body copes poorly with inflammation, and exogenous steroids are required. Therefore, pharmacogenetic polymorphism and single-nucleotide substitutions in the TSPO protein and steroidogenic enzymes must be taken into account as mutations can lead to inefficient NSs biosynthesis.

### 8.5. mPTP-Mediated Neuroprotection

In addition to TSPO inhibitors, the inhibitors of other mPTP components may have the potential to be used as neuroprotectors. mPTP is composed of a VDAC in the outer mitochondrial membrane, an adenine nucleotide translocator (ANT) in the inner mitochondrial membrane, and cyclophilin D (CypD) in the mitochondrial matrix, which is responsible for sensing the intracellular environment, oxidative stress, inflammatory cascade, pH imbalance, and ion disorders in response to tissue ischemia [167,168].

The modulation of mPTP is a mechanism of neuroprotection [10,169]. The role of mPTP in ischemic injuries, aging, and degenerative diseases and the agreement between in vitro molecular and structural studies and in vivo functional studies under physiological and pathological conditions are the topical problems of molecular pharmacology [170]. Preclinical studies include TSPO inhibitors, the blockers of cyclophilin D, and other components that form mPTP [93].

Among these are pharmacological substances already used in clinical practice; however, their indications for use are not related to neuroprotection (for example, the BCL-2 inhibitor venetoclax). The mPTP inhibitor cyclosporine is an immunosuppressant, mainly used to treat transplant recipients. Cyclosporine also has neuroprotective properties, inhibiting the opening of the mitochondrial pore; after traumatic brain injury in mice, cyclosporine reduces ROS production and restores the membrane potential of mitochondria. P4 itself, at particular doses, can selectively modulate mPTP. In the work of Fedotcheva and coauthors, a new synthetic analog of P4, 17a-acetoxy-3b-butanoyloxy-6-methyl-pregna-4,6-dien-20-one (butagest or gestobutanoyl), was compared to P4 and medroxyprogesterone acetate (MPA). P4 and butagest were found to have opposite effects on the induction of mPTP opening by calcium ions. MPA acted similarly to P4 but was less effective. Butagest inhibited the pore, while P4 and MPA stimulated pore opening. The inhibitory effect of butagest was eliminated in the presence of carboxyatractyloside, which selectively binds the thiol groups of adenylate translocase and prevents adenine nucleotide binding. These data suggest that butagest interacts with thiol groups [171]. The expression of ANT1 is also significantly regulated by steroid hormones. ANT isoforms in normal and cancerous cells could be new targets for the action of steroid hormones and anti-inflammatory drugs [44].

It was shown that mitochondria have sex-dependent functional features: the mitochondria of females show more efficient respiration and adenosine triphosphate (ATP) production as well as more effective antioxidant systems. The time of mPTP opening was greater in female rats than in male rats, which may be one of the reasons for the higher tolerance of females to ischemic injury. These differences may be caused by estradiol and P4, as they regulate mitochondrial respiratory activity [172].

The modulation of mPTP is a possible neuroprotective mechanism of P4. In male rats, P4 improves the rehabilitation parameters at a dose of 8 mg/kg body weight and acts as a neuroprotective modulator in cerebral ischemia: it reduces the mitochondrial ROS, attenuates the level of MAO, and lowers the activity of acetylcholine esterase in animals [169]. The use of P4 for males should be taken into account in future clinical trials.

P4 can act as cyclosporine, a well-known mPTP inhibitor [173]. Cyclosporine has been studied in clinical TBI trials. In a preclinical animal (porcine) 5-day dosing regimen with continuous intravenous infusion (20 mg/kg/day), cyclosporine reduced the volume of parenchymal injury by 35% and improved the profile of neuronal injury markers.

Cyclosporine is clinically used in transplant medicine for immunosuppression. It prevents the formation of ROS through the inhibition of mPTP and provides protection against oxidative damage. Many studies in TBI models have demonstrated the neuroprotective effects of cyclosporine [174]. A phase II study of the pharmacokinetics and safety of cyclosporine (NeuroSTAT) in patients with severe TBI revealed a tendency for a decrease in the level of several TBI biomarkers in the cerebrospinal fluid (NCT01825044, https://clinicaltrials.gov/study/NCT01825044 (accessed on 11 May 2025)) [16].

The synthetic analog of P4 gestobutanoyl (butagest) had an inhibitory effect on mPTP opening, acting like cyclosporine; it was well-tolerated and had a calming effect on rats, which holds promise for its future clinical application. Both gestobutanoyl and cyclosporine also inhibit P-glycoprotein; therefore, the SH groups of the nucleotide-binding domain of P-glycoprotein and the SH groups of ANT may be their common targets, which are attractive in neuroprotection.

## 9. Limitations and Prospects of DHEA and P4 Usage in Neuroprotection

The unreliability and limitations of animal experimentation often lead to the failures of new drugs in clinical trials because of disparities between animal models of disease and human diseases, species differences in physiology, and genetics [175]. Decades ago, it was shown that the levels of NSs increase sharply after exposure to various stressors in animal models [176]. The results of long-term studies on animals are extrapolated to clinical trials, but questions regarding the dose and the route of administration, as well as side effects, always arise. Preclinical studies of P4 demonstrated neuroprotection in animal models of toxic demyelination; P4 reduced brain infarct and improved motor and cognitive performance by neurogenesis, remyelination, and the activation of bioenergetics in mitochondria [17]. However, not all clinical trials of P4 as a neuroprotector have shown proven efficacy and positive results.

The possible reasons for the failures of P4 in some clinical trials are as follows: (1) the oral route of administration does not allow one to achieve the desirable concentration because of the low bioavailability of DHEA and P4; (2) a wide range of the age of the patients (for example, from 16 to 70 years) [11]; and (3) the lack of personalization based on the individual profile of the expression of sex hormone receptors, such as PR, AR, ER, and other neurosteroids receptors, and the activity of steroidogenic enzymes and transmembrane/mitochondrial transporters.

P4 showed benefits in women with cocaine use disorder [177] in the treatment of catamenial epilepsy [178] but did not show benefits in the treatment of TBI due to a very wide range of patient age, differences in sex, and heterogeneity of TBI [11]. Gender differences must also be taken into account: for example, ALLO caused sedation at a dose of 10 mg per day in women and at a dose of 6 mg per day in men [179]. The highest concentrations of ALLO are reached during the third trimester of pregnancy at levels up to 157 nmol/L, which are not associated with adverse effects for either the mother or the fetus [179]. For neuroprotection, some authors suggest more selective progesterone receptor ligands than P4, such as Nestorone [180,181], to avoid some P4-related side effects.

The clinical application of DHEA as a neuroprotector has still not been realized due to an inadequate dose regimen and the route of administration. Clinical trials demonstrated that oral 150 mg DHEA per day improved memory recollection and mood and decreased cortisol levels after a 7-day period [182], while 50 mg per day, twice a day, improved cognitive functioning only after 3 months of uptake [183].

In future clinical trials, it is desirable to use higher dosages of DHEA for oral uptake (above 100 mg per day) since the oral bioavailability of DHEA is about 1–3%, the percutaneous route increases the bioavailability to 33%, and the subcutaneous route increases bioavailability to 100%; however, the subcutaneous way of administration is not suitable, especially for elder patients [184]. Patches are comfortable, and skin penetration can be forced by transdermal drug delivery nanosystems, solid polymer-based ion-conductive porous microneedles [185], as well as synthetic and natural polymer hydrogels [186] and nanogels [187].

Great progress has been made in topical and transdermal drug delivery research based on nanotechnologies [188]; there are clinically approved formulations for testosterone, progesterone, and estradiol, but there is a lack of studies evaluating the tolerance of different pharmacological formulations of DHEA [189].

The prospects of clinical studies of NSs should be based on more concrete and inclusive criteria and outcome measures: the level of the expression of steroid hormone receptors, steroidogenic enzymes, mitochondrial proteins, as well as the pharmacogenetic testing of the polymorphisms of CYP450 isoforms, which markedly influence the pharmacodynamics of NSs. This is already starting to be implemented: in a current clinical trial of ALLO as a neuroprotector in AD (4 mg intravenous infusion, once per week, for a period of 12 months), ALLO was administered only to APOE ε4-positive AD patients aged 55 to 80 years.

## 10. Conclusions

Despite the large number of completed and ongoing clinical trials of neurosteroids as neuroprotectors, only a few have shown good clinical results, as evidenced by the emergence of new FDA-approved drugs such as brexanolone (Zulresso™) and zuranolone (Zurzuvae™) for the treatment of postpartum depression and ganaxolone (Ztalmy™) for the treatment of CDKL5 deficiency disorder. All of them are analogs of ALLO.

Brexanolone and zuranolone both demonstrated clinical effectiveness; brexanolone should be administered intravenously over a period of 60 h for approximately 2.5 days. Zuranolone treatment is much easier, with once-daily oral dosing; however, a potential for excessive sedation exists. DHEA derivatives are being tested. All of them are aimed at treating a particular pathology, as determined by clinical trials. However, for prophylactic anti-inflammatory, antihypoxic, antioxidant, and neuroregenerative effects, neurosteroids should be examined over a very long period of use, and the dose is very important. For prophylactic and rehabilitation outcomes, 50–300 mg/day DHEA or 8 mg/day P4 or ALLO is a desirable dosage based on the results of clinical trials. For acute injury conditions, higher dosages are preferred.

The molecular mechanisms of the neuroprotective action of P4, its metabolite ALLO, and DHEA are poorly understood. Their anti-inflammatory and stimulatory action toward nerve growth factors can play the main role in the treatment of neurodegenerative diseases. There is still no information on their exact effect on sigma receptor subtypes, CAR, PGRMC1, and the components of mPTP. Neurosteroids themselves and their derivatives capable of inhibiting the pore are promising candidates for neuroprotection. The megestrol acetate derivative gestobutanoyl (butagest), a pore inhibitor and P-glycoprotein inhibitor, may act as a potential neuroprotector.

It is very important to develop new goals and outcomes in clinical trials of NSs, such as target engagement, the evaluation of cerebrospinal fluid biomarkers, and the expression profiles of steroid receptors and steroidogenic enzymes. In animal models, including Zebrafish models, it is desirable to establish the clear functioning of NSs as activators or inhibitors of receptor-mediated signaling since the binding to the appropriate steroid receptor can be agonistic or antagonistic. Synthetic derivatives of P4 and DHEA have promise in this context as they can be more selective toward neuromodulating receptors. Due to the poor oral bioavailability of NSs, new formulations, such as nanogels or microneedle array patches, should be developed and clinically evaluated in randomized trials.

## Figures and Tables

**Table 1:** Clinically approved and investigational P4 and DHEA derivatives in neuroprotection.

Name	Chemical Structure	Condition/Disease	Ref.
DHEA and its derivatives			
DHEA	3beta-hydroxyandrost-5-en-17-one 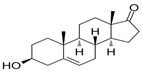	neonatal hypoxic–ischemic brain injury	[38]
Bezisterim (ne3107)	3α-ethynyl-androst-5-ene-3β,7β,17β-triol 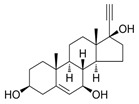	neuroinflammation and dementia	[39,40]
BNN27	17α,20R-epoxypregn-5-ene-3β,21-diol 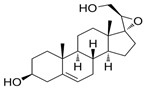	small-molecule mimetics of endogenous neurotrophin/Spinal Cord Injury	[41]
Fluasterone	3β-dehydroxy-16α-fluoro-DHEA 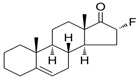	traumatic brain injury	[42]
P4 derivatives			
Megestrole acetate	17-Hydroxy-6-methylpregna-3,6-diene-3,20-dione 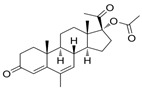	anorexia and cachexia or serious unexplained weight loss	[43]
Gestobutanoyl	17α-Acetoxy-3β-butanoyloxy-6-methyl-pregna-4,6-dien-20-one 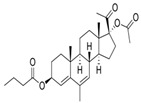	inflammation and MPTP opening inhibitor	[44]
PREG (eltanolone)			
Eltanolone (stereoisomer of 5α-pregnan-3α-ol-20-one (allopregnanolone)	3alpha-Hydroxy-5beta-pregnan-20-one 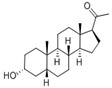	anesthetic/low back pain	[45,46]
ALLO and its derivatives			
Brexanolone (Zulresso™) or ALLO	5alpha-Pregnan-3alpha-ol-20-one 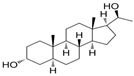	postpartum depression and Fragile X-Associated Tremor/Ataxia Syndrome	[47,48]
Ganaxolone (Ztalmy™)	3α-hydroxy-3β-methyl-5α-pregnan-20-one 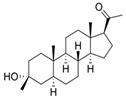	inhibition of epileptic seizures associated with cyclin-dependent kinase-like 5 deficiency disorder	[49]
Sepranolone	Isoallopregnanolone, 3-hydroxy-5alpha-pregnan-20-one 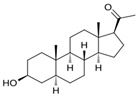	premenstrual dysphoric disorder	[50]
Zuranolone (*Zurzuvae*™, SAGE-217).	3beta-methyl-21-(4-cyano-1H-pyrazol-1′-yl)-19-norpregnanolone 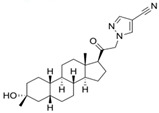	postpartum depression/major depressive disorder	[51,52,53,54]

## Data Availability

Not applicable.
